# Management of chest indrawing pneumonia in children aged 2–59 months by community-level workers compared to standard care on clinical outcomes: systematic review and meta-analysis

**DOI:** 10.7189/jogh.15.04169

**Published:** 2025-06-20

**Authors:** Barsha Gadapani Pathak, Yasir Bin Nisar, Uma Chandra Mouli Natchu, Rukman Manapurath, Archana Thakur, Sarmila Mazumder, Temsunaro Rongsen Chandola, Bireshwar Sinha

**Affiliations:** 1 Society for Applied Studies, New Delhi, India; 2 Department of Global Public Health and Primary Care, Centre for Intervention Science in Maternal and Child Health, Centre for International Health, University of Bergen, Bergen, Norway; 3 Department of Maternal, Newborn, Child and Adolescent Health and Ageing, World Health Organization, Geneva, Switzerland; 4 Department of Biotechnology, Wellcome India Alliance Clinical and Public Health Fellow, Hyderabad, India

## Abstract

**Background:**

World Health Organization recommends that children aged 2–59 months, with chest indrawing pneumonia, are treated with a five-day course of oral amoxicillin initiated at a health facility. We synthesised the evidence on the effectiveness and safety of community-level health care workers (CLHW)-led management compared to standard care at a hospital or health facility among children aged 2–59 months with chest indrawing pneumonia in terms of clinical outcomes.

**Methods:**

We conducted a comprehensive search in PubMed, EMBASE, and Cochrane Central for studies published from January 2000 to August 2024, with no language restriction. The search included quasi and/or randomised controlled trials. We conducted data extraction using the Cochrane checklist, and we used GRADE to assess the quality of the evidence. Given that the included studies were from different settings and used different methods, we applied a random-effects model for data synthesis. The primary outcome was treatment failure by six days of treatment initiation. Secondary outcomes included mortality and other serious adverse events (SAEs) (excluding mortality) by 14 days.

**Results:**

Of the 6883 eligible articles, we included three randomised controlled trials involving 11 779 children aged 2–59 months with chest indrawing pneumonia without danger signs. The CLHW-led management significantly reduced the risk of treatment failure by 34% (relative risk (RR) = 0.66, 95% confidence interval (CI) = 0.44, 0.98; low quality evidence). The RR of mortality for CLHW-led management compared to standard care was RR = 0.85 (95% CI = 0.31, 2.30; low quality evidence). The RR for other SAEs was RR = 1.20 (95% CI = 0.34, 4.26; low quality evidence).

**Conclusions:**

The findings suggest that CLHW-led management may reduce treatment failure rates by day six without any significant differences in mortality or other SAEs compared to standard care. However, given the low certainty of the evidence, these results should be interpreted with caution and more research is required

**Registration:**

PROSPERO: CRD42023439851.

Pneumonia is a leading cause of morbidity and mortality in children under five, accounting for approximately 784 600 deaths of the estimated 5.1 million deaths worldwide in children in 2021 [1]. The burden is higher in developing countries, with a global incidence rate of approximately 0.29 episodes per child yearly, leading to an astounding 151 million new cases annually [2]. Among these, 7–13% are severe enough to require hospital admission [2]. An estimated 22 million of the 138 million annual cases of pneumonia in children under five involve chest indrawing pneumonia [2]. Reducing delay in treatment initiation is critical to reduce mortality associated with childhood pneumonia [3,4]. Improving access to care by involving community-level health care workers (CLHWs) in the management of childhood pneumonia can be a crucial strategy in low-resource settings and difficult-to-reach areas [5].

According to the 2012 World Health Organization (WHO) guidelines and the updated classification for management of childhood pneumonia at health facilities, children with chest indrawing pneumonia should receive a five-day course of oral amoxicillin initiated at a health facility [6–8]. However, the available WHO guidelines have not provided specific recommendations for community-based management of chest indrawing pneumonia by CLHWs due to insufficient generalisable evidence, as most studies were limited to single-country settings [8]. Furthermore, understanding the effectiveness of community-level health care worker CLHWs interventions requires consideration of diverse contextual factors, including variations in local health care infrastructure, cultural beliefs and perceptions related to pneumonia, and training standards of health care workers. These contextual elements may significantly influence how interventions are implemented, accepted, and ultimately, their impact on health outcomes.

Recent evidence from two trials in low- and middle-income countries (LMIC), along with an implementation research study from Africa, has demonstrated the potential effectiveness of CLHWs in identifying and managing chest indrawing pneumonia in children aged 2–59 months without danger signs at the community level [9–11]. Furthermore, a multi-country cluster randomised control trial conducted in 2022, reported that CLHW-led management of chest indrawing without hypoxemia or any danger signs among children aged 2–59 months against standard care (as per national guidelines) had no substantial risk difference in treatment failure −1.0% (95% confidence interval (CI) = −3.0, 1.1) [12]. As per our knowledge, there are no existing systematic reviews or meta-analyses that have quantitatively pooled or synthesised the current available evidence on the impact of CLHW-led management on chest indrawing pneumonia among children aged 2–59 months at the community level to inform global policy.

To address this gap, we planned this systematic review and meta-analysis to evaluate the effectiveness of CLHW-led management compared to standard care on treatment failure in children aged 2–59 months with chest indrawing pneumonia with no danger signs. In addition, we compared the effect on safety outcomes (mortality, other serious adverse events). The pooled evidence will inform policy recommendations and may support updating management strategies for chest indrawing in children, particularly in resource-limited settings.

## METHODS

We performed a systematic search in the Cochrane Central Register of Controlled Trials, Cochrane Register of Studies Online, PubMed, and EMBASE (Box S1 in the **Online Supplementary Document**). We included randomised or quasi-randomised trials published from 2000 up to 31 August 2024. We included studies that compared community-based management of chest indrawing pneumonia, without any danger signs in children aged 2–59 months, with oral amoxicillin for five days provided by CLHWs *vs.* standard care, *i.e.* referral by CLHWs to a health facility or hospital-based management. Standard care in this review broadly refers to referral-based management of chest indrawing pneumonia. However, the specific approaches varied across studies depending on national guidelines and protocols (Box S2 in the **Online Supplementary Document**).

We excluded articles published before 2000 as they may not align with the current treatment protocol. Our search strategy had no language restrictions. For articles written in languages other than English, we reviewed and extracted data from the English abstract when available. For non-English articles without English abstracts, an online translation tool was used to screen and extract relevant data (Table S1 in the **Online Supplementary Document****)**.

Furthermore, CLHWs refer to trained lay or paraprofessional health personnel who deliver primary care services within the community. These workers operated within formal community-based programs such as integrated community case management or its national equivalent and received additional training, supervision, and support as part of the study interventions. In the absence of WHO-defined danger signs, we defined chest indrawing pneumonia as lower chest wall indrawing in a child aged 2–59 months with cough and/or difficulty breathing. While minor variations existed in how chest indrawing or danger signs were confirmed across studies, all studies followed WHO integrated management of childhood illnesses classification protocols. We harmonised these definitions during analysis to ensure consistency across study data.

### Outcomes

The primary outcomes were clinical deterioration and/or treatment failure among children aged 2–59 months with chest indrawing pneumonia after five days of treatment. The primary outcome of this review was treatment failure, defined broadly by included studies as either clinical deterioration or non-resolution of chest indrawing pneumonia according to WHO guidelines. Clinical deterioration was explicitly defined in individual studies as follows: appearance of danger signs such as inability to drink or breastfeed, convulsions, persistent vomiting, or lethargy, presence of very severe pneumonia symptoms, persistent fever with lower chest indrawing after 48 hours, or presence of fever or chest indrawing alone at day six and/or WHO-defined danger signs or hypoxemia (oxygen saturation <90%) along with persistent chest indrawing. While definitions varied slightly, we applied a harmonised operational definition, *i.e.* clinical deterioration (as defined by the study) and/or no resolution of chest indrawing pneumonia as per the WHO definition, to enable consistent pooling of outcome data. Secondary outcomes included mortality (all causes), and other serious adverse events (SAEs), excluding mortality. SAEs include serious anaphylactic reaction, severe diarrhoea, generalised severe rash, or any event that has led to changing or discontinuation of the study drug (Box S2 in the **Online Supplementary Document**).

### Study selection and data extraction

We used the Covidence systematic review software (Veritas Health Innovation, Melbourne, Victoria, Australia) to review the data [13]. Two authors independently screened titles and abstracts to identify relevant citations based on predefined inclusion criteria. We conducted full-text reviews for selected articles. We extracted data using a modified version of the Cochrane Effective Practice and Organisation of Care group data collection checklist from the Cochrane Effective Practice and Organisation of Care Group in London, UK, and Northern Ireland [14]. This checklist covered various details, including study identifiers, context, design, intervention specifics, treatment dose and duration, and study outcomes. Any disagreements or discrepancies between reviewers were resolved through discussion or by consulting a third author.

### Data analysis

We followed the guidelines outlined in the Cochrane Handbook for Systematic Reviews of Interventions for data analysis [15]. We reported pooled relative risks (RRs) along with 95% CIs for categorical variables. We employed a random-effects model using the restricted maximum likelihood ratio to combine data and estimate effects. The random-effects model is suitable and commonly recommended for meta-analysis involving studies conducted in diverse contexts because it effectively captures variability both within and between studies. Therefore, we chose this model given the anticipated methodological diversity and differences in implementation methods/approaches across the included studies [15]. We planned to employ Egger’s test to evaluate publication bias for outcomes reported in at least five studies [16]. Additionally, to comprehensively explore heterogeneity among the included studies, we conducted a post-hoc subgroup analysis for the primary outcome based on study location (Asia *vs.* Africa) and the implementation strategy used to identify participants for referral. We performed these exploratory analyses in response to the high observed heterogeneity, and we did not pre-specify them in the protocol.

We evaluated the risk of bias in the studies we included by using the revised Cochrane risk-of-bias tool for randomised trials [14,17]. We conducted a quality assessment using the GRADE approach to evaluate the certainty of the pooled estimates [18]. We used the PRISMA guideline to report all the findings [19].

## RESULTS

### Meta-analysis results

We identified 6883 records (Figure S1 in the **Online Supplementary Document**), of which we selected 42 articles. Finally, three relevant randomised trials from five LMICs were eligible for inclusion in our meta-analysis. These reported on the effect of community-based management by CLHWs *vs.* standard care on chest indrawing pneumonia without danger signs in 11 779 children aged 2–59 months (**Table 1**) [10–12]. The risk of bias assessment showed some concerns in all three trials. Primarily, we found all three trials to have a potential bias in the measurement of outcomes. All trials demonstrated low risk of bias in the domains related to randomisation, deviations from intended interventions, missing outcome data, and selective reporting (Figure S2 in the **Online Supplementary Document**).

**Table 1:** Description of the studies and summary statistics for the effect of managing chest indrawing pneumonia without danger signs among children aged 2–59 mo by community-based management by CLHWs *vs.* standard care on study outcomes

Study and country	Study design	Population	Intervention	Control	Sample size (n)	Outcome	Effect size (95% CI)
Soofi et al. 2012 [11], Pakistan	Cluster randomised trial	Children aged 2–59 mo having suspected pneumonia were screened by LHWs, *i.e*. CLHWs and classified as WHO-defined pneumonia without danger signs	Children with pneumonia without danger signs were prescribed oral amoxicillin syrup (90 mg/kg per day in two doses) by LHWs for 5 d at home	One dose of oral co-trimoxazole and were referred to their nearest health facility for admission and intravenous antibiotics, as per government policy	4410	Primary: treatment failure at 6 d	RD = 5.2% (95% CI = 13.7, 3.3)
Bari et al. 2013 [10], Pakistan	Cluster-RCT	Children aged 2–59 mo with pneumonia without danger signs *i.e.* lower chest indrawing, regardless of respiratory rate in children with a history of cough and/or difficult breathing	LHWs provided mothers with oral amoxicillin (80–90 mg/kg/d in 2 divided doses) for 5 d with specific guidance on its use	LHWs gave the first dose of oral cotrimoxazole and referred to a health facility for appropriate treatment, which was standard care	3472	Primary: treatment failure at 6 d	RD = 8.9% (95% CI = –12.4, 5.4) by day 6
EMPIC 2022 [12], Africa and Asia	Cluster randomised trial	Children aged 2–59-mo with chest indrawing without hypoxemia or any danger signs	Community-based treatment group: treat with oral amoxicillin (50 mg/kg body weight per dose) twice a day for 5 d without referral to a facility by CLHWs. Refer children with danger signs to the hospital (after the first dose of antibiotic). Conduct pulse oximetry on children with pneumonia and refer hypoxemic children to the hospital, which supervisors will confirm	Facility-based treatment group: referral to a health facility after 1st dose of antibiotics	3897	Primary: treatment failure at 6 d. Secondary: 1) feasibility of using a pulse oximeter by CLHWs, 2) performance of CLHWs for using pulse oximetry against a standard measurement by a trained study supervisor, 3) the impact of pulse oximetry on referral and treatment outcomes	RD = –0.01 (95% CI = −1.5, 1.5)

CI – confidence interval, CLHWs – community level health care workers, EMPIC – enhanced management of pneumonia in community, LHW – lady health workers, RD – risk difference, RCT – randomised control trial

The operational definition of standard care varied across studies. In the study by Soofi et al., standard care was defined as administering a single dose of oral co-trimoxazole by the CLHWs, followed by referral to the nearest hospital for admission and intravenous antibiotics [11]. In the study by Bari et al., standard care was defined as administration of one dose of co-trimoxazole and immediately referring the child to the nearest health facility for further management. In cases of refused referral to a health facility/hospital, home treatment was offered with co-trimoxazole for five days [10]. In the Enhanced Management of Pneumonia in Community (EMPIC) trial, standard care involved immediate referral of a child with chest indrawing pneumonia to a first-level facility for further management, following the standard integrated community case management protocol [12]. The EMPIC trial [12] specifically included cases of chest indrawing pneumonia without danger signs and hypoxemia. The hypoxemia was verified using a pulse oximeter in EMPIC trial, while the other two trials [10,11] excluded cases that had the presence of any danger signs or severe malnutrition identified by clinical examination. The danger signs included inability to drink or breastfeed, convulsions, persistent vomiting or vomiting everything, or abnormally sleepy or difficulty waking. In the EMPIC study, the dosage is 50 mg/kg, leading to a total of 100 mg/kg per day. In the other two studies, the dosage is 90 mg/kg per day, divided into two doses of 45 mg/kg each [10–12].

### Primary outcomes

**Figure 1:**
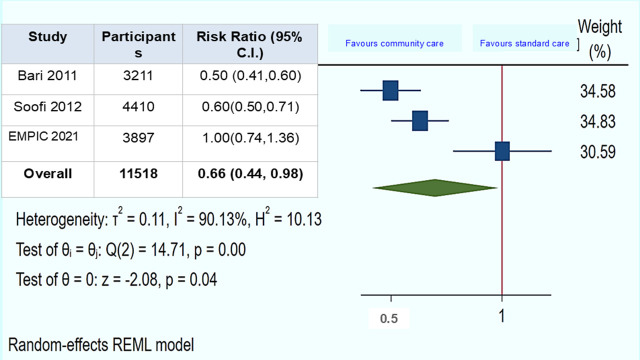
Community-based management by CLHWs *vs.* standard management: treatment failure/clinical deterioration among children with chest indrawing pneumonia assessed on the sixth day.

In the included children with chest indrawing pneumonia (n = 11 518) across three trials, the pooled RR of treatment failure of community-based management by CLHWs compared to standard care assessed at six days after enrolment was RR = 0.66 (95% CI = 0.44, 0.98, *I^2^* = 90.13, *P* = 0.04) (**Figure 1**). A few children (n = 261) were excluded from the analysis due to loss to follow-up, protocol violations, or withdrawal of consent. This outcome was low certainty of evidence due to concerns regarding risk of bias and significant heterogeneity (reflected by high *I^2^* values).

### Secondary outcomes

**Figure 2:**
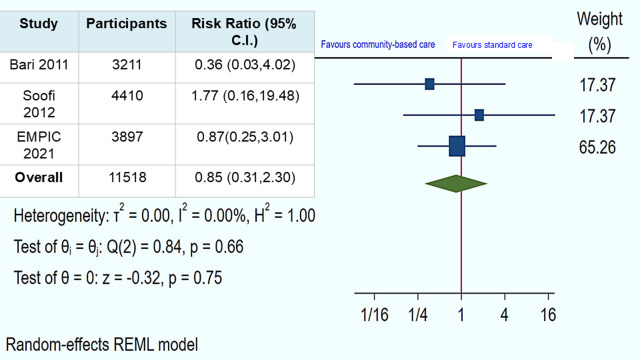
Community-based management by CLHWs *vs.* standard management/care: mortality among children with chest indrawing pneumonia was assessed on the 14th day.

**Figure 3:**
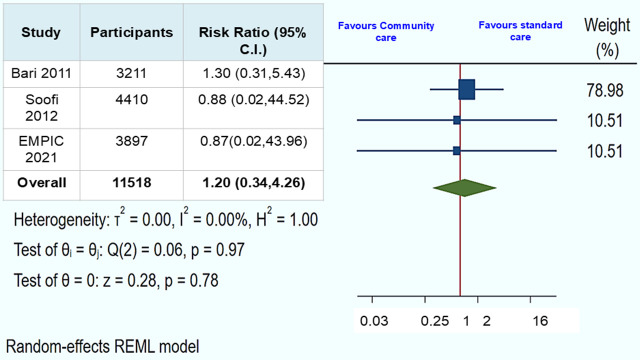
Community-based management by CLHWs *vs.* standard care: other serious adverse events among children with chest indrawing pneumonia were assessed on the 14th day.

In the included children with chest indrawing pneumonia (n = 11 518) across three trials, the pooled RR estimate for mortality in the community-based management group by CLHWs *vs.* the standard care group assessed at 14 days from enrolment was RR = 0.85 (95% CI = 0.31, 2.30, *I^2^* = 0.00, *P* = 0.75) (**Figure 2**). The pooled RR of other serious adverse events (excluding mortality) assessed at the same time point was RR = 1.20 (95% CI = 0.34, 4.26, *I^2^* = 0.00, *P* = 0.78) (**Figure 3**). The mortality and serious adverse events outcomes were low certainty of evidence due to concerns regarding risk of bias, and a wide level of confidence interval (**Table 2**).

**Table 2:** GRADE assessment in children 2–59 mo of age with chest indrawing and no general danger signs, what is the effectiveness of management done by community-level health workers (using oral amoxicillin) compared to standard care in improving clinical outcomes at all levels of care

Certainty of evidence assessment							Patients, n/N (%)		Effect (95% CI)		Certainty	Importance
**Studies (n)**	**Study design**	**Risk of bias**	**Inconsistency**	**Indirectness**	**Imprecision**	**Other considerations**	**Community-based care**	**Standard care**	**Relative**	**Absolute**		
Treatment failure at day 6 (n = 3)*	Randomised trials	Serious†	Serious‡	Not serious	Not serious	None	434/6279 (6.9)	587/5239 (11.2)	0.66 (0.44, 0.98)	38 fewer per 1000 (from 63 fewer to 2 fewer)	Low	Critical
Mortality at day 14 (all cause) (n = 3)	Randomised trials	Serious†	Not serious	Not serious	Serious§	None	8/6279 (0.1)	8/5239 (0.2)	0.85 (0.31, 2.30)	0 fewer per 1000 (from 1 fewer to 2 more)	Low	Critical
Other serious adverse effects (n = 3)	Randomised trials	Serious†	Not serious	Not serious	Serious§	None	5/6279 (0.1)	3/5332 (0.1)	1.20 (0.34, 4.26)	0 fewer per 1000 (from 0 fewer to 2 more)	Low	Critical

CI – confidence interval, RR – risk ratio

*Clinical deterioration (as defined by the study) and/or no resolution of chest indrawing pneumonia as per the WHO definition).

†Downgraded two levels as three studies had some concerns in risk of bias assessment.

‡Downgraded one level as *I^2^* value 90.13% and *P* = 0.00.

§Downgraded one level due to a wide CI.

¶Serious anaphylactic reaction, severe diarrhoea, generalised severe rash, events that required a change of therapy or discontinuation of therapy. This does not include mortality.

A post-hoc subgroup analysis by place of study showed that RR for treatment failure in the children with chest indrawing pneumonia of community-based management group by CLHWs *vs.* standard care chest indrawing pneumonia in the Asian sites (n = 9822) across three trials was RR = 0.66 (95% CI = 0.43, 1.00, *I^2^* = 89.64, *P* = 0.00) and that in the African sites (n = 1696, one trial) was RR = 0.93 (95% CI = 0.61, 1.43, *I^2^* = 0.00, *P* = 1.00). The findings in both regions were not statistically significant (Figure S3 in the **Online Supplementary Document**).

Additionally, a post hoc subgroup analysis based on the implementation strategy used for selecting the participants for referral indicated that trails using clinical assessment for assessing hypoxemia (n = 7621) across two trials showed a reduced risk of treatment failure with community-based care (RR = 0.55; 95% CI = 0.46, 0.66, *I^2^* = 49.38, *P* = 0.16) while the trial which employed pulse oximetry for assessing hypoxemia (n = 3897) demonstrated no difference of treatment failure between the community-based management group by CLHWs *vs.* standard care chest indrawing pneumonia (RR = 1.00; 95% CI = 0.74, 1.36, *I^2^* = 0.00, *P* = 1.00).

## DISCUSSION

We included three trials from five LMICs of Africa and Asia, which enrolled 11 779 children (aged 2–59 months) to evaluate the effect of community-based management of chest indrawing pneumonia cases without danger signs by CLHWs compared to the standard care. We found low-quality evidence indicating a significant 34% lower risk of treatment failure at day six of treatment initiation in the community-based management (with oral amoxicillin) group by CLHWs compared to the standard care group. There was no substantial difference in the risk of death and other serious adverse events on day 14 from treatment initiation in the community-based management group compared to the standard care group.

Several earlier reviews support that oral amoxicillin is as effective as injectable penicillin for the management of pneumonia in standard or inpatient care. However, no previous reviews have summarised the effect of managing uncomplicated chest indrawing pneumonia with oral amoxicillin by CLHWs in a community setting [20,21]. In 2010, results from a systematic review including 10 studies and approximately 10 000 participants showed that community case management of pneumonia could potentially reduce pneumonia mortality by up to 70% with a moderate certainty of evidence [22]. A cohort study from Bangladesh further indicates that treating pneumonia without danger signs in local community-based health facilities is not associated with a higher risk of treatment failure compared to referral and admission to hospitals [23]. The successful implementation of a pragmatic trial in Kenya, where CLHWs effectively managed chest indrawing pneumonia, highlighted that CLHWs can effectively treat pneumonia even in high-mortality settings [9]. Literature from across the globe has demonstrated that CLHWs are increasingly effective in addressing system vulnerabilities, specifically by alleviating workforce shortages [5,24].

During the formulation of the current WHO pneumonia management recommendation, only two randomised controlled trials from Pakistan showed that properly trained and supported community health workers can effectively and safely treat chest indrawing pneumonia at home with oral amoxicillin [6–8]. However, no quantitative pooled analysis was performed since both studies were from Pakistan, and thus, no specific WHO recommendation exists for the community-based management of chest indrawing pneumonia by CLHWs [8]. In 2021, the EMPIC group conducted a multi-country cluster-randomised, open-label non-inferiority trial in rural areas of Bangladesh, Ethiopia, India, and Malawi. They found that non-hypoxemic children with chest indrawing pneumonia without any danger signs treated with a five-day course of oral amoxicillin by trained, equipped, and supervised CLHWs had similar outcomes compared to the currently recommended facility-based treatment. In our meta-analysis, we included all available randomised controlled trials to date and corroborated the earlier evidence that for the treatment of chest indrawing pneumonia in children aged 2–59 months, community-based management by CLHWs compared to standard care can possibly lead to reduced treatment failure and comparable clinical outcomes. We conducted a post-hoc exploratory subgroup analysis by geographic region to investigate potential contributors to the high heterogeneity observed in the treatment failure outcome. While this analysis suggests that geographic variation may partly explain the heterogeneity, the findings should be interpreted with caution as the analysis was not pre-specified. Managing childhood diseases through properly trained and supervised community-level staff is feasible, leads to quicker care, and is as effective, safe, and less costly compared to referrals and treatments in health facilities [5,24]. Furthermore, in 2012 Salim et al., conducted a cost-effective analysis of a randomised controlled trial and reported that the average household cost per case for a CLHW-managed case was USD 1.46, compared to USD 7.60 for referred cases as per the standard care protocol [25]. Excluding the cost of antibiotics provided by the CLHW program, the cost per case was USD 0.25 for community-managed cases by CLHWs and USD 7.51 for referred cases, indicating a 30-fold difference [25].

Our meta-analysis findings show that community-based management by CLHWs results in comparable or better clinical outcomes than standard care for chest indrawing pneumonia and are both plausible and policy-relevant. The success of CLHWs in managing this condition can be attributed to their ability to detect illness early, provide immediate intervention, and ensure timely management. Rapid support and assistance from CLHWs in their communities can significantly benefit the caregivers of under-five children. This strategy might effectively circumvent treatment delays, particularly in difficult-to-reach areas, simplify health care access for families from a familiar source, thereby contributing to successful case management as well as being cost-effective [25–27]. Further, equipping these health care workers with tools such as pulse oximeters could enhance correct case detection rates and expedite their decision-making process for case referrals, particularly in instances of hypoxemia. This approach, implemented in one of the trials included in our review, has been reported as a feasible strategy [12].

While involving CLHWs in childhood pneumonia management holds promise, several challenges need to be addressed to ensure scalability and effectiveness. A significant hurdle lies in sustaining CLHWs competency, especially in areas with infrequent encounters with sick children and limited educational qualifications. Implementing regular capacity-building sessions, refresher training, and supportive supervision can mitigate this challenge. Pulse oximeters offer the potential for CLHWs to identify hypoxemic cases requiring prompt referral. However, concerns exist regarding device quality, the appropriate selection of probe sizes for different age groups, and ensuring these tools complement, not replace, clinical assessments. While one trial conducted in an LMIC setting reported a 91% correct performance by CLHWs in pulse oximetry, further research is warranted to assess its widespread use [12]. Additionally, mortality rates in community-based management by CLHWs were consistently low, remaining below 1% across all three trials. An important consideration when interpreting our findings is the inherent variability in health care infrastructure, cultural attitudes towards pneumonia, and the standards of training provided to CLHWs across the diverse study settings. Differences in these contextual factors could explain some of the heterogeneity observed in our pooled analysis. Future studies and planned implementation research [28] may systematically document these aspects to better understand their role and facilitate the design of contextually appropriate strategies for pneumonia management.

Children with lower chest indrawing may have non-bacterial pneumonia, which does not always require antibiotic treatment [29,30]. Unfortunately, LMICs lack an effective point-of-care test to differentiate between viral and bacterial infections [12]. It is possible that the administration of antibiotics by CLHWs might raise concerns about antimicrobial resistance and may need monitoring [29]. Nonetheless, community-based management by CLHWs using oral amoxicillin for chest indrawing pneumonia offers several benefits. It can mitigate risks such as complications from injections, including needle-borne infections, unnecessary referrals or hospital admissions, and the use of higher second-line antibiotics [11]. Children receiving intravenous antibiotics for pneumonia are at risk of bloodstream infections, particularly with suboptimal catheter care. Hospitalised children may also acquire nosocomial infections or multidrug-resistant organisms, complicating recovery [31,32] Hence, treating these children in the community can reduce the likelihood of health care-associated infections, which are more common in hospital settings. Furthermore, the CLHW-led management strategy in LMICs might possibly reduce the need to visit private or unregistered practitioners and avoid irrational antibiotic use [33]. As with any medical intervention, the success of community-based management by CLHWs depends on context-specific factors, including resource availability and local health care infrastructure.

### Study implications

These findings have important practical implications for policy and programmatic actions, particularly in low-resource settings. Successful implementation of community-based management for chest indrawing pneumonia without danger signs requires tailored approaches, including structured and ongoing training programs for CLHWs, consistent supportive supervision, and secure logistics for drug supply. Furthermore, introducing technologies such as pulse oximetry to improve diagnostic accuracy and timely referral practices, coupled with community awareness campaigns to foster local understanding and acceptance, can enhance program effectiveness. Policymakers and program managers should consider these context-specific strategies to optimise community-based pneumonia management.

### Limitations and strengths

All the trials included in the review had ‘some concerns’ in bias assessment. These biases could impact the validity of the conclusions drawn. Although no studies were excluded due to language limitations in this review, as reflected in the PRISMA flow diagram (Figure S1 in the **Online Supplementary Document**), the use of translation tools for non-English articles may carry a potential risk of misinterpretation of information. There are also some heterogeneities in the selection of the cases for the studies. One of the included studies employed the use of pulse oximeters to identify hypoxemic cases and excluded children with danger signs, and/or with hypoxemia. While in the other two trials, CLHWs identified danger signs based on clinical signs and symptoms, which seem to correlate with hypoxemia, and referred those children to an appropriate health facility. Nonetheless, irrespective of the approach used to screen children, the treatment failure rates were either not much different or lower in the community-based management group by CLHWs compared with standard care (Figure S4 in the **Online Supplementary Document**). The findings from this review primarily stem from LMICs with community-level health care worker programs and may not apply to other high-income countries. While our findings indicate potential benefits of community-level management for chest indrawing pneumonia in reducing treatment failures, the evidence presented is of low certainty due to methodological concerns and substantial heterogeneity among included trials. Therefore, caution is warranted when interpreting these results. Before broadly recommending community-level health care worker-led interventions, it is crucial to conduct further high-quality randomised controlled trials and robust implementation research to validate these findings, clarify contexts under which this strategy is most effective, and address the existing evidence gaps. We emphasise that further research, including implementation research in program settings and evaluation, is warranted to improve our understanding.

In the review, we conducted a comprehensive search across multiple databases, ensuring a robust inclusion of relevant studies. The collective participation of 11 779 participants across the included trials provides substantial statistical power for assessing primary and secondary outcomes. Rigorous quality assessment using the GRADE approach of the pooled evidence enhances the utility of the findings. Although the risk of confounding was inherently minimised by including randomised controlled trials, residual heterogeneity arising from differences in study settings, participant populations, and intervention implementations remains a limitation. However, we conducted subgroup analyses based on geographical regions and explored implementation aspects to better understand and address this heterogeneity.

## CONCLUSIONS

Low-certainty evidence from three randomised controlled trials with 11 518 participants in LMICs countries in Africa and Asia showed that community-based management by CLHWs of chest indrawing pneumonia in children aged 2–59 months with a five-day course of oral amoxicillin significantly reduces treatment failure by 34% compared to standard care. Mortality and other serious adverse events showed no significant differences between the groups. These findings support that involving the existing CLHWs for chest indrawing pneumonia management in low-resource settings may potentially improve clinical outcomes for children aged 2–59 months, possibly through early identification, prompt treatment, and reducing referral-related delays. Nonetheless, the findings are to be interpreted with caution, given the low certainty evidence and should be contextualised. Training and supportive supervision for CLHWs may be important.

## Additional material


Online Supplementary Document
