# Supplementary material for: Management of chest indrawing pneumonia in children aged 2–59 months by community-level workers compared to standard care on clinical outcomes: systematic review and meta-analysis
Source: J Glob Health. 2025 Jun 20;15:04169. doi: 10.7189/jogh.15.04169 (PMC12178591; doi:10.7189/jogh.15.04169)
Supplement: Online Supplementary Document [file jogh-15-04169-s001.pdf]

Supplement to: Gadapani Pathak B, Nisar YB, Natchu UCM, Manapurath R, Thakur A, Mazumder S, Chandola TR, Sinha B. Management of chest indrawing pneumonia in children aged 2–59 months by community-level workers compared to standard care on clinical outcomes: systematic review and meta-analysis. J Glob Health. 2025;15:04169.

Details:

eBox 1: Search strategy for Community-based management done by community-level health workers compared to standard management in improving clinical outcomes among under-fives with chest indrawing pneumonia

eFigure 1: PRISMA: Community-based management by community-level healthcare worker versus standard management

eFigure 2: Risk of bias assessment for the included studies

eFigure 3: Post-hoc subgroup analysis based on study settings (African versus Asian settings): Community-based care by community-level health workers (CLHWs) versus standard management: Treatment failure/Clinical deterioration among children with chest indrawing pneumonia.

eFigure 4: Post-hoc subgroup analysis based on the strategy used for selecting the participants for referral: Community-based care by community-level health workers (CLHWs) versus standard management: Treatment failure/Clinical deterioration among children with chest indrawing pneumonia assessed on the 6th day.

eTable 1: Certain relevant articles which has been excluded from review with reasons

eBox 2: Operational Definitions:

**Box 1**: Search strategy for Community-based management done by community-level health workers compared to standard management in improving clinical outcomes among under-fives with chest indrawing pneumonia.		
**PUBMED**		
**Search block**	**Terms**	**Hits on 18/07/2024**
#1 (Disease)	("pneumonia"[All Fields]) OR (pneumon*[tw]) OR ("bronchopneumonia"[All Fields]) OR ("pleuropneumonia"[All Fields]) OR ("fast breathing pneumonia"[All Fields]) OR (difficult breathing [tiab]) OR ("tachypnea"[All Fields]) OR ("chest indrawing pneumonia"[All Fields]) OR ("bronchiolitis"[All Fields])	374,412
#2 (Population, only 0-5 years)	((((((((((Infant[MeSH Terms]) OR (infant*[Title/Abstract])) OR (infancy[Title/Abstract])) OR (newborn*[Title/Abstract])) OR (baby*[Title/Abstract])) OR (babies[Title/Abstract])) OR (neonat*[Title/Abstract])) OR (preterm*[Title/Abstract])) OR (prematur*[Title/Abstract])) OR (child*[Title/Abstract])) OR ("under-five"[All Fields]) OR ("under"[All Fields] AND "five*"[All Fields]) OR (preschool*[Title/Abstract])	3,086,883
#3 (Medicines)	((((((((((((Antibiotics [MeSH Terms]) OR (Anti-bacterial agent*[MeSH Terms])) OR (Anti-infective agent*[MeSH Terms])) OR (Penicillins [MeSH Terms])) OR (antibiotics[MeSH Terms])) OR (antibacterial*[Text Word])) OR (anti-bacterial[Text Word])) OR (bacteriocid*[Text Word])) OR (antimicrobial[Text Word])) OR (antimicrobial*[Text Word])) OR (antiinfective*[Text Word])) OR (anti-infective*[Text Word])) OR (amoxicillin[MeSH Terms])	1,096,410

#4 (Study Design)	("cohort studies"[MeSH Terms] OR "cross sectional studies"[MeSH Terms] OR "randomized controlled trial"[Publication Type] OR "controlled clinical trial"[Publication Type] OR "clinical trial"[Publication Type] OR "Randomized"[Title/Abstract] OR "placebo"[Title/Abstract] OR "clinical trials as topic"[MeSH Major Topic] OR "randomly"[Title/Abstract] OR "Trial"[Title] OR "case control studies"[MeSH Terms] OR "observational studies"[Text Word] OR "observational studies"[Title/Abstract] OR "Preprint"[Publication Type])	4,781,210
#5 (Publication types)	(Address*[ptyp] OR Autobiography[ptyp] OR Bibliography[ptyp] OR Biography[ptyp] OR pubmed books[filter] OR Case Reports[ptyp] OR Congress*[ptyp] OR Consensus Development Conference[ptyp] OR Directory[ptyp] OR Duplicate Publication[ptyp] OR Editorial[ptyp] OR Festschrift[ptyp] OR Guideline[ptyp] OR Interview[ptyp] OR Lecture*[ptyp] OR Legal Case*[ptyp] OR News[ptyp] OR Newspaper Article[ptyp] OR Personal Narrative*[ptyp] OR Portrait*[ptyp] OR Retracted Publication[ptyp] OR Twin Study[ptyp] OR Video-Audio Media[ptyp])	3,831,932
#6 Search block for Q3 and Q4	# 1 AND # 2 AND # 3 AND #4	**3697**
#7 (FINAL FOR 3 &4)	#6 NOT #5, Filter humans ONLY	**3655**

**EMBASE**	
**Search Block**	**Terms**
Disease	('pneumonia'/exp OR 'pneumonia':ab,ti OR 'bronchopneumonia':ab,ti OR 'pleuropneumonia':ab,ti OR 'fast breathing pneumonia':ab,ti OR 'difficult

	breathing':ti,ab OR 'tachypnea':ab,ti OR 'chest indrawing pneumonia':ab,ti OR 'bronchiolitis':ab,ti)
	**AND**
Population, only 0-5 years	(((((((((('Infant'/exp OR infant:ab,ti OR infancy:ab,ti OR newborn:ab,ti OR baby:ab,ti OR babies:ab,ti OR neonat*:ab,ti OR preterm:ab,ti OR prematur*:ab,ti OR child:ab,ti) OR 'under-five':ab,ti) OR ('under' AND 'five*'):ab,ti) OR preschool:ab,ti)
	**AND**
Medicines	(((((((((('Antibiotic'/exp OR 'Anti-bacterial agent'/exp OR 'Anti-infective agent'/exp OR 'Penicillins'/exp OR antibiotics:ab,ti OR antibacterial*:ab,ti OR 'anti-bacterial':ab,ti OR 'bacteriocid*':ab,ti OR antimicrobial:ab,ti OR antimicrobial*:ab,ti OR antiinfective*:ab,ti OR 'anti-infective':ab,ti OR amoxicillin:ab,ti)
	**AND**
Study Design	(('cohort studies'/exp OR 'cross sectional studies'/exp OR 'randomized controlled trial'/exp OR 'controlled clinical trial'/exp OR 'clinical trial'/exp OR 'Randomized':ab,ti OR 'placebo':ab,ti OR 'clinical trials as topic'/exp OR 'randomly':ab,ti OR 'Trial':ti OR 'non randomized controlled trial*':exp OR 'case control studies'/exp OR 'observational studies':ti,ab OR 'Preprint':ab,ti)
	**NOT**
Publication types	(Address*:ptyp OR Autobiography:ptyp OR Bibliography:ptyp OR Biography:ptyp OR 'pubmed books':ftyp OR 'Case Reports':ptyp OR 'Congress*':ptyp OR 'Consensus Development Conference':ptyp OR Directory:ptyp OR 'Duplicate Publication':ptyp OR Editorial:ptyp OR Festschrift:ptyp OR Guideline:ptyp OR Interview:ptyp OR 'Lecture*':ptyp OR 'Legal Case*':ptyp OR News:ptyp OR 'Newspaper Article':ptyp OR 'Personal Narrative*':ptyp OR 'Portrait*':ptyp OR 'Retracted Publication':ptyp OR 'Twin Study':ptyp OR 'Video-Audio Media':ptyp)
Total hits (**18.08.2024)**	**2515**

Cochrane library	
Search	For CENTRAL, we used the pre-identified MeSH terms “Pneumonia” and “drug therapy-DT” with no limitations for language and date of publication.<br><br>MeSH descriptor: [Pneumonia] this term only and with a qualifier(s): [drug therapy - DT
Total hits (18.08.2024)	**713** (date:10/8/2023)

Figure 1: PRISMA: Community-based management by community-level healthcare worker Versus standard management

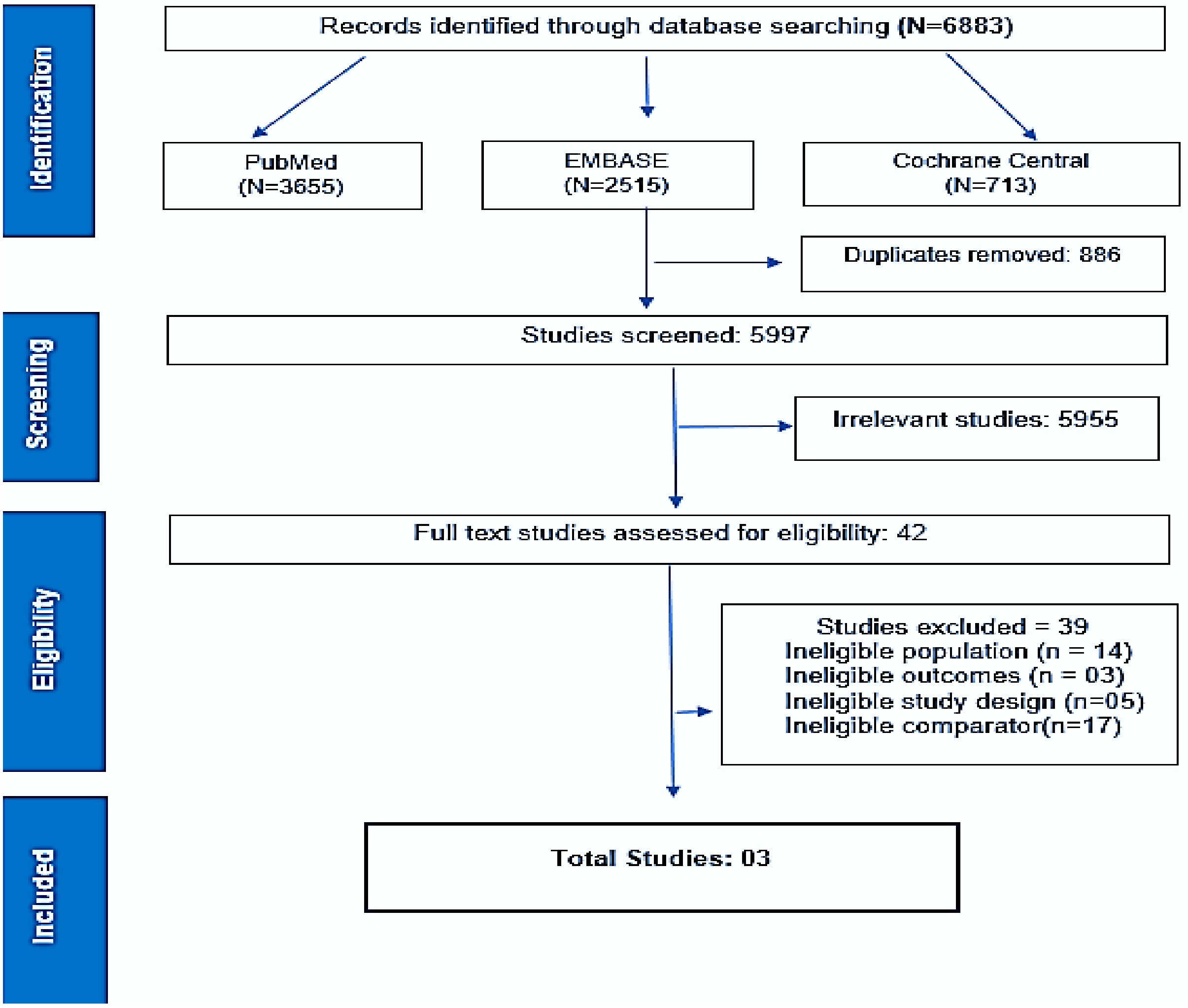

Figure 2: Risk of bias assessment for the studies

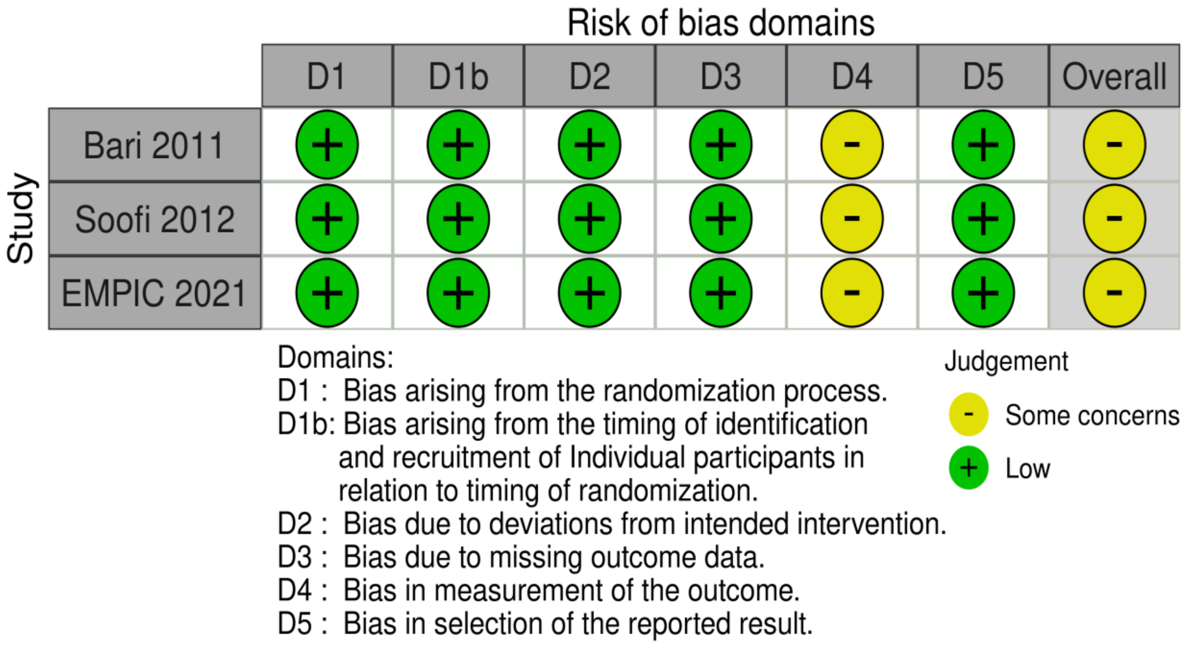

Figure 3: Post-hoc subgroup analysis based on study settings (African versus Asian settings): Community-based care by community-level health workers (CLHWs) versus standard management: Treatment failure/Clinical deterioration among children with chest indrawing pneumonia assessed on the 6th day.

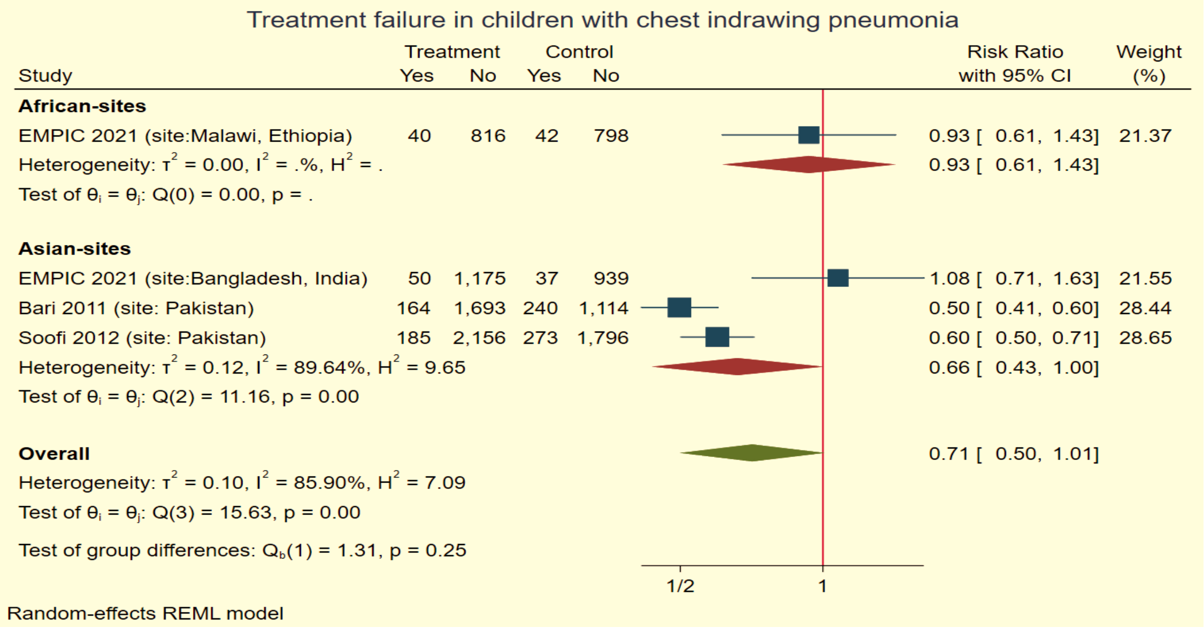

Figure 4: Post-hoc subgroup analysis based on the strategy used for selecting the participants for referral: Community-based care by community-level health workers (CLHWs) versus standard management: Treatment failure/Clinical deterioration among children with chest indrawing pneumonia assessed on the 6th day.

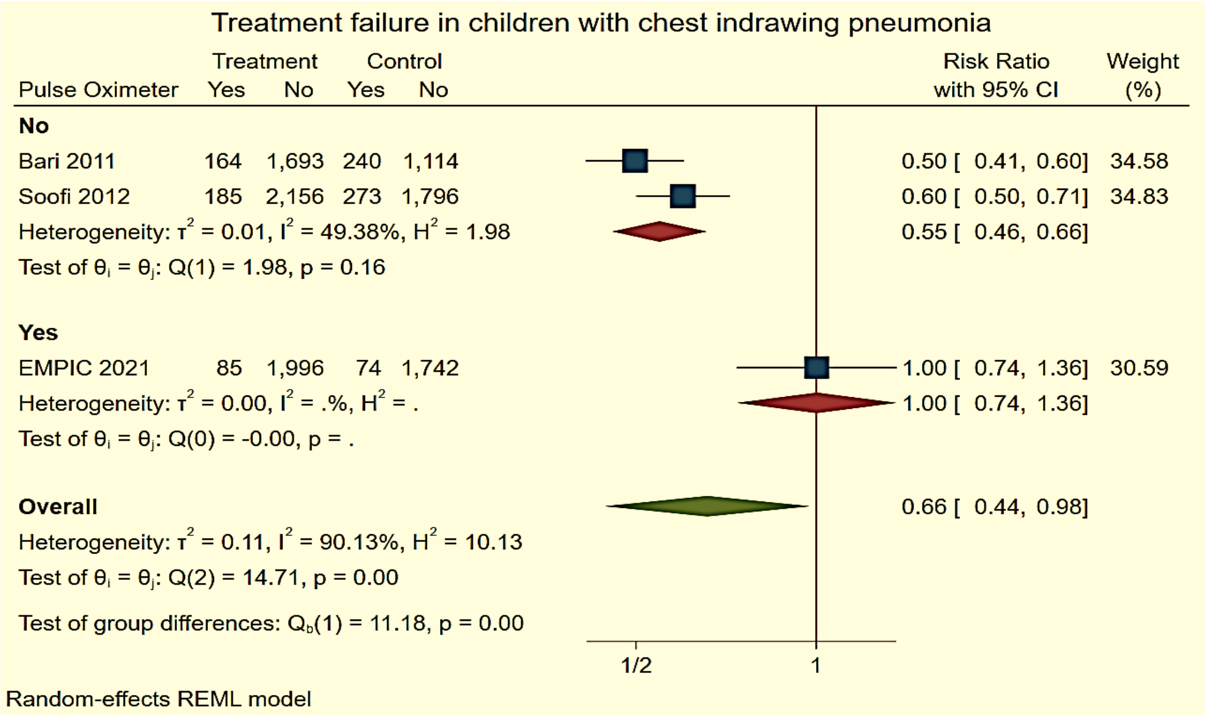

Table 1: Certain relevant articles which has been excluded from review with reasons				
**Author, year**	**Intervention**	**Control**	**Outcome**	**Reason for non-inclusion in review**
Hazir 2008	**Ambulatory group**: Children received the first dose of oral amoxicillin from their caregiver under supervision of the study physician and were then sent home to complete a 5-day course.	**Hospitalised group**: Children were admitted for 48 h. Children who showed improvement in the first 48 h were discontinued from parenteral antibiotic treatment and received oral amoxicillin for another 3 days (80–90 mg/kg per day in two doses) at home.	-Treatment failure up to or on day 6.<br>-Treatment failure/Relapse 7-14 days	-The intervention is not completely provided by the community healthcare workers.<br><br>-The care provided in the control group seems to be different from the national guideline/standard care guidelines
Addo Yobo,2004	Oral amoxicillin to severe pneumonia cases (fast breathing and/or chest indrawing without danger signs)	Parenteral penicillin to severe pneumonia cases	Treatment failure (persistence of lower chest indrawing or new danger signs) at 48 h.	-It’s a hospital-based study.<br>- No community healthcare staff is involved it delivery of intervention.

Addo Yobo,2011 (MASS study)	Inclusion criteria: Oral amoxicillin to severe pneumonia cases without any danger signs		Treatment failure (persistence of lower chest indrawing or new danger signs) at 48 h.	- Multi-centre observational study<br>-Oral Amoxicillin is provided by the primary caregiver and there is no mention of involvement of community healthcare worker
Atkinson, 2007, PIVOT trial	Oral amoxicillin for community acquired pneumonia in children (PIVOT trial):	Intravenous benzyl penicillin for community acquired pneumonia in children	Treatment failure	-This study has included community acquired pneumonia and not defined pneumonia as per the WHO guideline, hence excluded<br>-Hospital-based study<br>-No community healthcare staff were included intervention group.

Box 2: Operational Definitions:

Treatment Failure (by day of outcome assessment) defined by any one of:

- Clinical deterioration (as defined by the study) or,
- No resolution of chest indrawing pneumonia as per WHO definition.

Study-wise operational definitions

Study	Definition of clinical deterioration.
Bari_2011	Appearance of a danger sign (unable to drink/breastfeed, convulsions, vomits everything, abnormally sleepy/difficult to wake), Temperature ≥100 °F, and lower chest indrawing.
Soofi_2012	The appearance of any signs of very severe pneumonia, the persistence of fever greater than 38°C with lower chest indrawing on day 3 (after 48 h of initiation of treatment), Either fever greater than 1000 F or lower chest indrawing alone at day 6
EMPIC_2021	Presence of a danger sign (WHO-defined) or SpO2 <90%, and persistence of chest-indrawing.

Study	Definitions of Serious adverse events
EMPIC 2021	serious anaphylactic reaction, severe diarrhea, or generalized severe rash.
Bari 2011	Events that required a change of therapy.
Soofi 2012	serious adverse events related to amoxicillin that necessitated discontinuation or change of the study drug.

Standard care: The standard management protocol followed by the country for management of under-five pneumonia with chest-indrawing

**Study**	**Definitions of standard care**

EMPIC 2021	The children with chest indrawing pneumonia were immediately sent to a referral facility after giving one dose of oral amoxicillin by the Community level healthcare staff for further management as per the standard protocol which is based on integrated management of childhood illness (IMCI) and integrated community case management (iCCM) protocol.
Bari 2011	Community level healthcare staff gave first dose of oral cotrimoxazole and referred to a health facility for appropriate treatment, which was standard of care.
Soofi 2012	Children were given one dose of oral co-trimoxazole (for children aged 2–11 months, sulfamethoxazole [200 mg] plus trimethoprim [40 mg]; for children aged 12 months to 5 years, sulfamethoxazole [300 mg] plus trimethoprim [60 mg]) by community level healthcare staff and referred to their nearest health facility for admission and intravenous antibiotics, as per the government policy and standard of care.

**Study**	**Definitions of community level healthcare workers (CLHWs)**
EMPIC 2021	In this multi-country study, CLHWs included cadres such as Accredited Social Health Activists (ASHAs) in India and their equivalents in other settings. These workers operated within existing integrated community case management (iCCM) programs and were trained to identify signs of pneumonia, use pulse oximeters to detect hypoxemia, administer a first dose of oral amoxicillin, and refer eligible children to health facilities. Their role focused on early identification, stabilization, and referral.
Bari 2011	*Community-level health workers* were **Lady Health Workers (LHWs)** under Pakistan's National Program for Family Planning and Primary Health Care. These workers were trained to identify signs of pneumonia and refer cases to health facilities. In this study, LHWs administered the first dose of co-trimoxazole and referred children with chest indrawing pneumonia for standard facility-based care. The LHWs received additional training as part of the study.

Soofi 2012	CLHWs were **Lady Health Workers (LHWs)** from the Pakistan's National Program for Family Planning and Primary Health Care. These LHWs received enhanced training in pneumonia assessment and classification per WHO guidelines and were responsible for both identifying and managing cases of chest indrawing pneumonia at home using oral amoxicillin. They provided treatment, follow-up visits, and adverse event monitoring.
